# Rising challenges in periprosthetic joint infections: a focus on rare pathogens and their clinical implications

**DOI:** 10.3389/fcimb.2024.1451398

**Published:** 2024-12-04

**Authors:** Jianhua Lyu, Jiagu Huang, Jiexin Huang, Hongxin Hu, Qijin Wang, Haiqi Ding, Hongyan Li, Xinyu Fang, Wenming Zhang

**Affiliations:** ^1^ Department of Orthopaedic Surgery, the First Affiliated Hospital, Fujian Medical University, Fuzhou, China; ^2^ Department of Orthopaedic Surgery, Affiliated Hospital of Putian University, Putian, China; ^3^ Department of Orthopaedic Surgery, National Regional Medical Center, the First Affiliated Hospital, Fujian Medical University, Fuzhou, China; ^4^ Fujian Provincial Institute of Orthopaedics, the First Affiliated Hospital, Fujian Medical University, Fuzhou, China; ^5^ Department of Orthopaedic Surgery, Ningde Municipal Hospital of Ningde Normal University, Ningde, China; ^6^ Department of Orthopaedic Surgery, Nanping First Hospital Affiliated to Fujian Medical University, Nanping, China; ^7^ Department of Orthopaedics, Affiliated Mindong Hospital of Fujian Medical University, Fuan, China

**Keywords:** periprosthetic joint infection, revision, microbiology, rare pathogen, next-generation sequencing

## Abstract

**Objectives:**

The study aimed to evaluate differences in clinical characteristics and treatment outcomes of periprosthetic joint infection (PJI) attributed to rare versus common pathogens.

**Methods:**

Data on PJI patients who underwent hip or knee arthroplasty at our center from April 2013 to December 2022 were retrospectively collected. Among the 219 enrolled patients, we compared 32 cases of PJI caused by rare pathogens with 187 controls of PJI caused by common pathogens, analyzing demographic information, clinical characteristics, and treatment outcomes.

**Results:**

In demographic data, the Charlson comorbidity index and preoperative invasive procedures were identified as risk factors for rare pathogen PJI. Clinically, the rare pathogen cohort exhibited a significantly higher rate of sinus tract formation compared to those with common bacteria PJI. In terms of laboratory findings, the mean serum C-reactive protein (CRP) was significantly lower in the rare pathogen group. This cohort also had a significantly lower culture positivity rate and a higher rate of polymicrobial co-infections. The median hospital stay was statistically longer for rare pathogen PJI cases than for those with common bacteria PJI. Furthermore, the rare pathogen group required longer antibiotic treatments and had higher rates of antibiotic-related adverse events, although reinfection rates did not significantly differ.

**Conclusion:**

PJI caused by rare pathogens exhibits distinct clinical presentations. With advances in diagnostic techniques such as metagenomic next-generation sequencing (mNGS), optimized culture methods, and an interdisciplinary approach facilitating early targeted treatment, rare pathogen PJIs may achieve outcomes comparable to those of typical cases.

## 1 Introduction

Total joint arthroplasty effectively enhances the quality of life for patients with severe joint diseases (Walker et al., 2002; Learmonth et al., 2007; Bumpass and Nunley, 2012), with a survival rate of over 95% for hip and knee prostheses exceeding 10 years post-surgery (Kapadia et al., 2016). Despite this success, periprosthetic joint infection (PJI) is a significant complication, affecting long-term implant survival and carrying an estimated 1.5-2% risk of development within 15 years post-surgery, alongside a concerning 20% mortality rate within five years of diagnosis (Patel, 2023). PJI not only inflicts physical and mental suffering but also imposes a substantial burden on families and society (Premkumar et al., 2021). While *Staphylococcus aureus* and coagulase-negative *staphylococci* are the primary PJI pathogens, accounting for 50-60% of infections (Rafiq et al., 2006), the incidence of PJI caused by rare pathogens is growing. These rare pathogens present with atypical clinical features, complicating diagnosis and often leading to worse outcomes. A study identified rare pathogens in 9.7% of PJIs (Anagnostakos et al., 2021), highlighting the need for increased awareness and improved diagnostic strategies.

As joint replacement utilization increases with our aging population, correspondingly, the incidence of PJI cases caused by rare pathogens is anticipated to rise. Diagnosing and treating these rare pathogen PJIs presents significant challenges (Koutserimpas et al., 2021; Chisari et al., 2022), often due to the lack of a comparative control group in existing studies. To bridge this knowledge gap, we conducted a retrospective analysis of patients treated for hip and knee PJI at our institution. Our goal was to identify the clinical presentation, optimal diagnostic strategies, and effective postoperative infection control for PJI caused by rare pathogens, and to contrast these with features and outcomes of infections caused by common bacteria.

## 2 Materials and methods

### 2.1 Patient selection

This retrospective case-control study was approved by our Institutional Review Board. Data of patients treated for hip or knee PJI at our institution between April 2013 to December 2022 were retrospectively analyzed. The PJI diagnosis was made collectively by an orthopedic surgeon, a microbiologist, and an infectious disease specialist based on the Musculoskeletal Infection Society (MSIS) criteria. Patients who underwent revision surgery for PJI at our hospital and had complete documentation were included. Exclusion criteria included: 1) PJI patients underwent joint replacement due to bone tumors; 2) PJI patients with incomplete medical documentation; 3) PJI patients followed up for less than one year; 4) PJI patients without identified pathogens.

### 2.2 Identification of pathogens

In instances where microbial cultures were negative yet metagenomic next-generation sequencing (mNGS) outcomes were positive, and scenarios where cultures indicated single bacterial infections in contrast to mNGS indicating multiple, criteria were applied to ascertain if the mNGS findings represented “true-positives,” as supported by existing literature (Wang et al., 2024).

### 2.3 Clinical data collection

For all enrolled cases, data on demographic details, medical history, clinical presentation, laboratory findings, surgical and antibiotic treatment, durations of antibiotic therapy, antibiotic-related adverse events, lengths of stay, and reinfection rates were extracted. Reinfection refers to the recurrence of an infection with the same pathogen or the introduction of a different pathogen, resulting in a new infection at the original site. For patients undergoing mNGS, results were recorded. Demographics of the study population included gender, age, smoking status, body mass index (BMI), Charlson Comorbidity Index (CCI) (Charlson et al., 2022), surgical joint, and preoperative invasive procedures. These preoperative invasive procedures, including joint aspiration, catheterization, and endotracheal intubation, were conducted before the index surgery. Laboratory results comprised white blood cell (WBC) count, preoperative C-reactive protein (CRP) and erythrocyte sedimentation rate (ESR), synovial white blood cell (WBC) count, polymorphonuclear percentage (PMN%), culture positivity and polymicrobial rates. Clinical manifestation includes acute/chronic onset, sinus tracts. Outcomes encompassed surgical strategies, hospitalization duration, follow-up length, antibiotic treatment duration, antibiotic-related adverse events, and PJI relapse.

### 2.4 Definitions

The diagnosis of PJI was based on standard Musculoskeletal Infection Society criteria (Parvizi et al., 2011a). Pathogens were defined as “rare” based on criteria from the research performed by Anagnostakos et al (Anagnostakos et al., 2021). Specifically, organisms were considered rare causes of periprosthetic joint infection if: 1) Uncommonly reported in association with PJI;2) Described in 10 or fewer English language case reports/small case series; 3) *Fungal* organisms. By this definition, a rare organism is either an atypical cause of PJI or has limited documentation in the literature. This includes classic pathogens with low relative PJI frequency as well as emerging bacteria and *fungi* with minimal prior evidence supporting their role in prosthetic infections. If cases exhibit polymicrobial infections involving both common and rare pathogens, with the rare pathogens being the primary infectious agents, they are categorized under the rare pathogen PJI group for the purposes of this study.

### 2.5 Surgical strategies

All operations were performed by the same surgical team under general or spinal anesthesia. The Tsukayama classification system guided treatment decisions. For acute hematogenous or postoperative infections without sinus tracts (Tsukayama Type II/III), debridement with implant retention (DAIR) was typically performed. One-stage revision arthroplasty was chosen for candidates with Tsukayama Type I/IV without sinus, assuming infecting organisms were not multi-drug resistant based on preoperative aspiration. One-stage revision was also favored for elderly patients with substantial medical comorbidities. Two-stage revisions were reserved for chronic Tsukayama Type IV infections with multidrug-resistant organisms on aspiration and compromised soft tissue envelopes, particularly with sinus tract formation. Ultimately, patient preferences also influenced the final approach.

### 2.6 Follow up

Patients were followed via clinic visits and phone calls, with reinfection and death as endpoints. They underwent routine serum testing, which included regular reexamination of ESR and CRP levels, as well as assessments of liver and renal function. These tests were conducted at specific intervals (3 months, 6 months, and 1 year post-operation) and then annually, with a minimum follow-up period of 2 years. The Delphi consensus definition was utilized to define infection control (Diaz-Ledezma et al., 2013). By these stringent criteria, infection control requires both complete eradication of the isolate based on clinical, microbiologic, and operative findings, while surviving through the treatment course without infection-associated complication or reoperation (Malekzadeh et al., 2010). This definition ensures durable elimination of infection rather than transient suppression alone.

Antibiotic-related adverse events were classified as (Xu et al., 2022): 1) Myelosuppression, defined as pretreatment white blood cell count over 4×10^9^/L declining to below 3×10^9^/L during intravenous or oral therapy; 2) Hepatotoxicity, indicated by 1.5-fold increase in peak aspartate aminotransferase or alanine aminotransferase above baseline normal pretreatment values; 3) Nephrotoxicity, determined by greater than 1.5-fold increase in serum creatinine over baseline normal pretreatment level; These categories identify antibiotic-associated toxicity based on suppression of hematopoietic cell lines, liver function, and renal function using established laboratory thresholds signifying organ damage attributable to administered antimicrobials.

### 2.7 Statistical analysis

Statistical analysis was performed using SPSS 20.0 and GraphPad Prism 8.0.2. Continuous data were first assessed for normality. Normally distributed variables were expressed as mean ± standard deviation and compared with the t-test, while non-normally distributed variables were presented as median (interquartile range) and compared using the Mann-Whitney U test. Categorical data were evaluated with the Chi-square or Fisher’s exact tests. Reinfection over time was analyzed by Kaplan-Meier methodology. Statistical significance was defined as *P <*0.05. Potential risk factors were screened from baseline demographic and clinical data. Independent variables with *P <*0.2 were incorporated into a binary logistic regression model to identify independent predictors of rare pathogen PJI.

## 3 Results

### 3.1 Demographic characteristics

**Figure 1:**
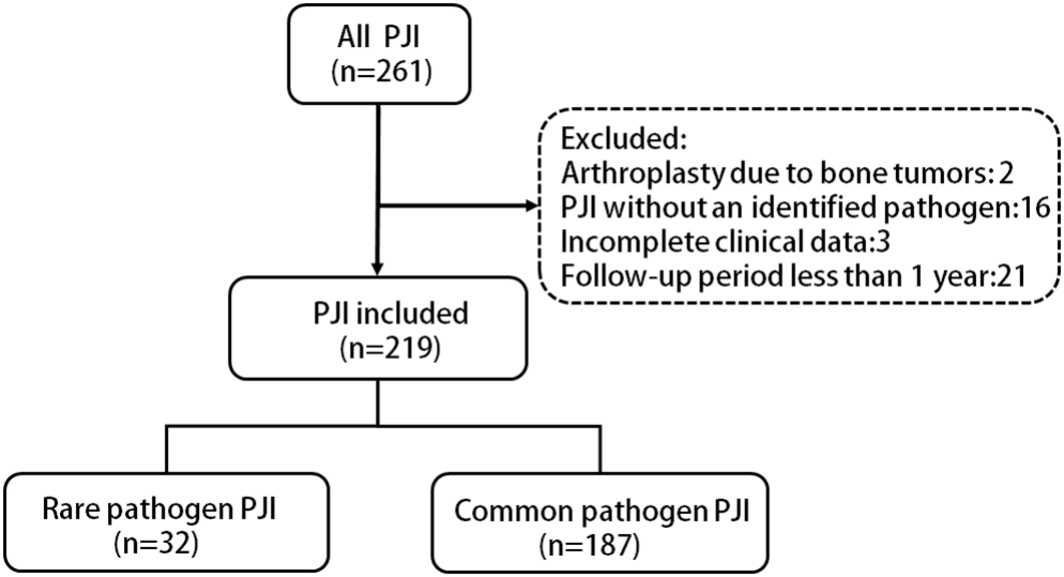
Flow chart of the inclusion, exclusion and grouping of PJI cases in this study.

**Figure 2:**
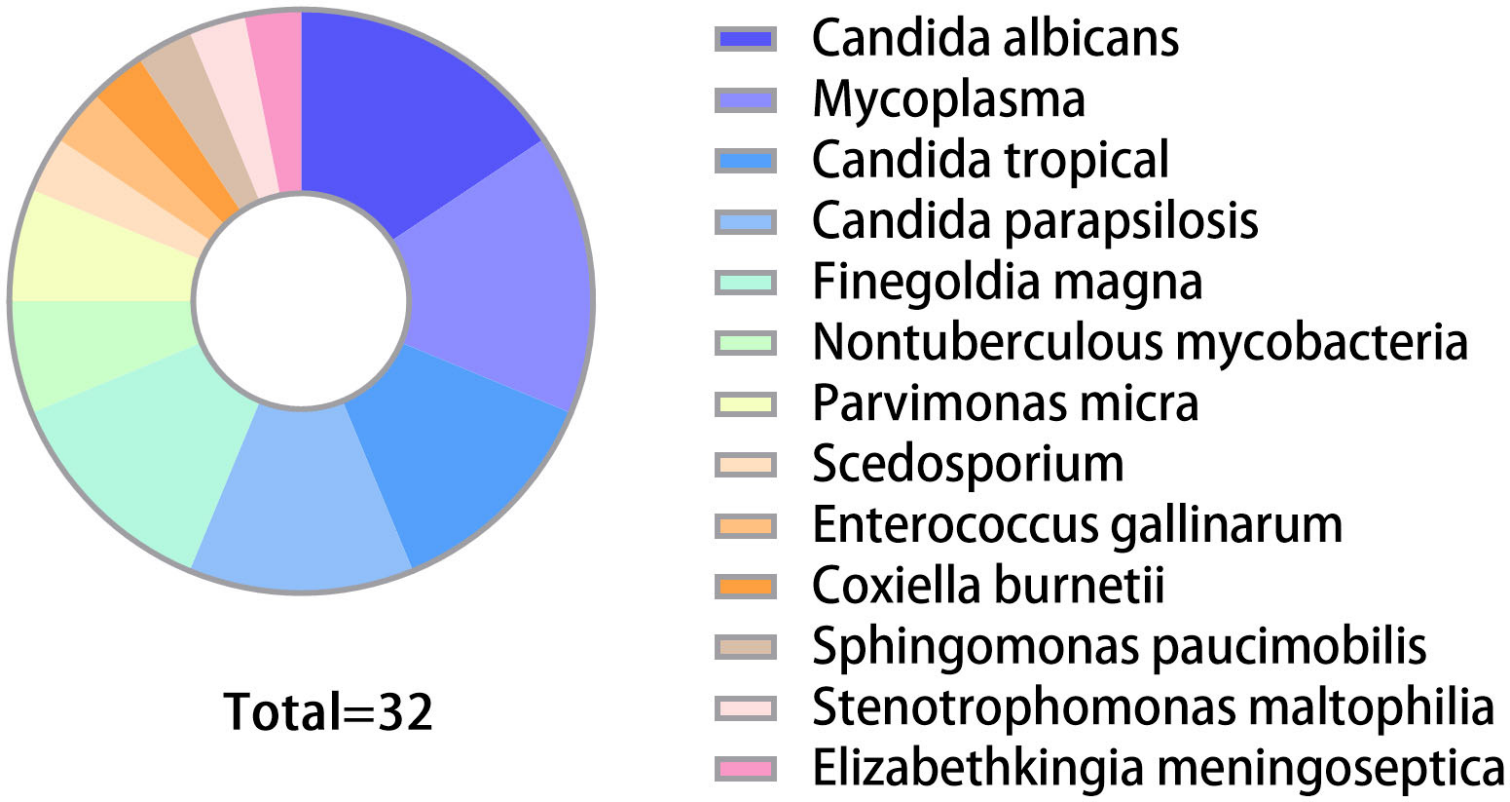
Proportions of various rare pathogens in the group. The group included 5 cases each of Candida albicans and Mycoplasma. There were 4 cases each of Candida tropical, Candida parapsilosis, and Finegoldia magna. Nontuberculous mycobacteria and Parvimonas micra each accounted for 2 cases. Additionally, there was 1 case each of *Scedosporium, Enterococcus gallinarum, Coxiella burnetii, Sphingomonas paucimobilis, Stenotrophomonas maltophilia*, and *Elizabethkingia meningoseptica*.

261 patients treated for hip or knee PJIs at our institution from April 2013 to December 2022 were identified. After applying exclusion criteria, 219 patients were included (**Figure 1**). Of these, 32 (14.6%) rare pathogen PJI cases were compared to 187 (85.4%) common pathogen PJI controls. The distribution of rare pathogens is shown in **Figure 2**. Baseline characteristics were similar between groups, with no statistical differences in age, gender, BMI, surgical site, or smoking status (**Table 1**). Higher CCI and proportion of preoperative invasive procedures were more common in the rare pathogen cohort, differences that were statistically significant. Indeed, binary logistic regression modeling identified CCI and preoperative invasive procedures as independent predictors of rare pathogen PJI.

**Table 1:** Comparison of variables between rare pathogens and common pathogens groups.

Variables	Rare Pathogens (n=32)	Common Pathogens (n=187)	*P*-value	Logistic regression *P*-value
Age (yrs)	65.6 ± 12.7	65.4 ± 11.3	0.9557^a^	
Gender (male/female)	11/21	81/106	0.4387^c^	
BMI (kg/m^2^)	25.3 ± 3.1	24.4 ± 3.2	0.1463^a^	0.071
Joint (hip/knee)	15/17	104/83	0.8412^b^	
Smoking (y/n)	7/25	56/131	0.4047^c^	
CCI	3.1 ± 1.4	2.4 ± 1.4	0.0049^a^	0.006
PIP (y/n)	26/6	103/84	0.0173^c^	0.029
Tsukayama Type [(II or III)/IV]	5/27	26/161	0.7855^c^	
Sinus tract (y/n)	14/18	46/141	0.032^c^	
Laboratory data				
WBC (×10^9^/l)	6.5 ± 2.3	7.6 ± 5.2	0.2451^a^	
CRP (mg/l)	28.2 ± 28.5	44.3 ± 44.1	0.0475^a^	
ESR (mm/h)	63.1 ± 33.8	62.1 ± 33.8	0.8846^a^	
SF WBC (/ml) (interquartile range)	8788 (3591, 13941)	9107 (4544, 30032)	0.1944^d^	
SF PMN (%)	76.1 ± 19.3	74.9 ± 16.4	0.7004^a^	
Culture positive rate (%)	53.1	73.8	0.0216^c^	
Polymicrobial infection rate (%)	25	9.1	0.0155^c^	

a Independent-samples t-test. Quantitative data are expressed as mean ± standard deviation.

b Chi-squared test.

c Fisher’s exact test.

d Mann-Whitney U test.

BMI, body mass index; CCI, Charlson comorbidity index; PIP, preoperative invasive procedures; WBC, white blood cell count; CRP, C-reaction protein; ESR, erythrocyte sedimentation rate; SF, synovial fluid; PMN, polymorphonuclear neutrophils.

### 3.2 Comparison of clinical variables

Clinically, the rare pathogen cohort had a significantly higher rate of sinus tract formation compared to common bacteria cases. Specifically, sinus tracts were observed in 5/15 *fungal* PJIs, 3/5 *mycoplasma* PJIs, 2/4 *Finegoldia magna* PJIs, and notably, 2/2 *non-tuberculous mycobacteria* cases. Regarding laboratory findings, mean serum CRP was lower in the rare pathogen group, a statistically significant difference. While mean WBC count also trended lower in rare cases, this difference did not reach statistical significance. No significant differences existed in ESR, synovial WBC or PMN%. The rare pathogen cohort had a significantly lower culture positivity rate (53.1%) compared to common bacteria controls (73.8%). Additionally, polymicrobial co-infections were more common with rare pathogens, affecting 25% of cases versus only 9.1% of common bacteria PJIs, a statistically significant difference.

**Figure 3:**
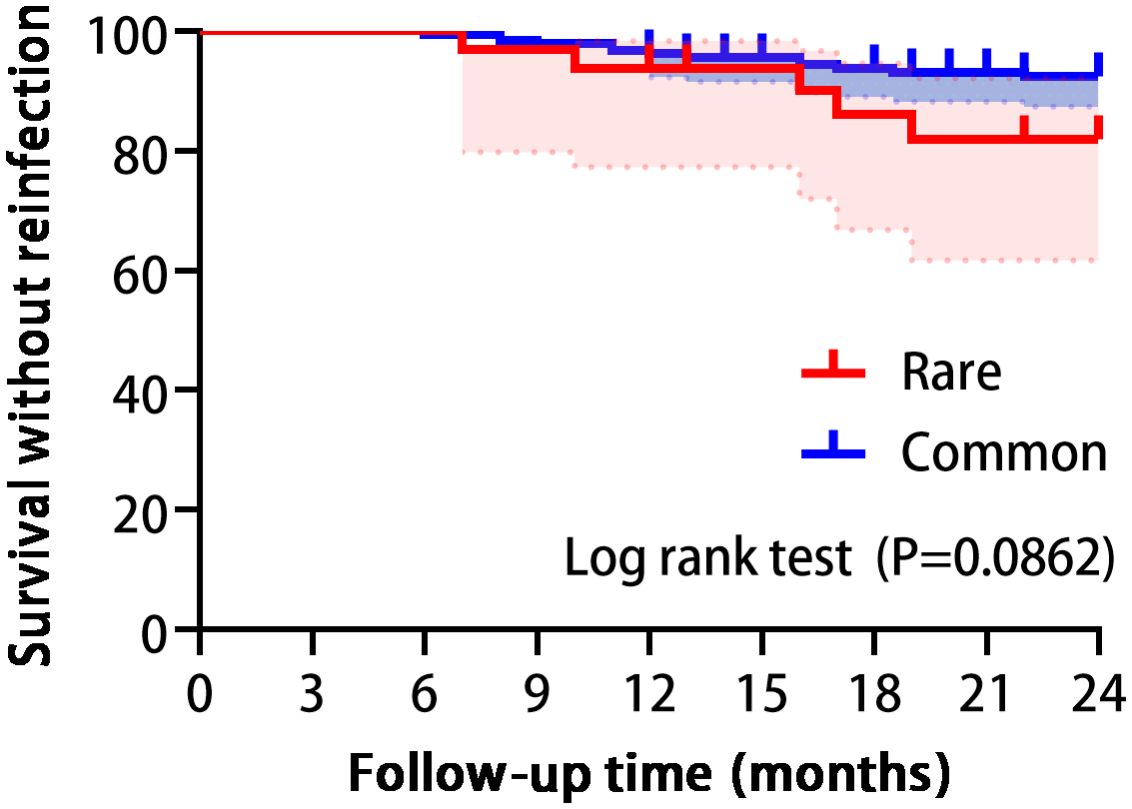
Survival analysis showed no statistically significant difference between the two groups in reinfection. (Log-rank P=0.0862).

Among the 219 patients, median follow-up and treatment approaches did not significantly differ between groups. However, rare pathogen PJI cases had a statistically longer median hospital stay compared to common bacteria controls. Additionally, the rare group required longer courses of antibiotic therapy and experienced higher rates of antibiotic-related adverse events. Notably, the one-year reinfection rates were 6.3% for the rare pathogen group and 3.7% for the common pathogen group. Furthermore, over the two-year period, these rates increased to 15.6% and 7.5%, respectively. Nevertheless, the 2-year reinfection rate was not significantly different between cohorts, as confirmed by Kaplan-Meier analysis (**Table 2**, **Figure 3**). All cases of reinfection underwent a two-stage revision surgery to further eradicate the infection.

**Table 2:** Comparison of outcomes between rare pathogens and common pathogens groups.

Variables	Rare Pathogens n=32	Common Pathogens n=187	*P*-value
Follow-up period (months)	40.8±21.2	42.5±23.8	0.7164^a^
Treatment			0.2792^b^
DAIR	2	32	
One-stage revision	10	56	
Two-stage revision	20	99	
Length of hospital stay (days)	22.2±5.0	18.7±5.8	0.0013^a^
Duration of antibiotic use (days)	151.6±50.4	88.3±25.1	<0.0001^a^
Antibiotic complications (y/n)	8/24	18/169	0.0325^c^
Reinfection (y/n)	5/27	14/173	0.1664^c^

a Independent-samples t-test.

b Chi-squared test.

c Fisher’s exact test.

DAIR, Debridement, antibiotics and implant retention.

## 4 Discussion

In evaluating and managing PJI, rare pathogen causes will inevitably be encountered. In prior studies, PJIs caused by rare organisms ranged from 4.1-9.7% (Rafiq et al., 2006; Tsai et al., 2019; Anagnostakos et al., 2021). In our current series, 14.6% of PJIs resulted from rare pathogens, including 8.3% from *fungi* and *mycobacteria*. This indicates that the incidence of rare pathogen PJI varies among centers, and its presence should not be overlooked clinically.

The rare pathogen cohort had a significantly higher polymicrobial infection rate than common bacteria PJIs, which demonstrates that rare organisms are more likely to participate in polymicrobial infections. Indeed, prior studies confirm higher rates of multiple organisms in PJI with sinus tracts (Marculescu and Cantey, 2008; Li et al., 2021), which were also more prevalent in our rare pathogen group. As external communications, sinus tracts likely enable invasion and propagation of multiple bacteria, including rare species. Diagnosis and treatment of polymicrobial PJI is notoriously challenging, with worse outcomes compared to monomicrobial infections (Marculescu et al., 2006; Parvizi et al., 2011b; Osmon et al., 2013). In a prior analysis, Mei et al. found combining mNGS with conventional cultures enhanced detection of polymicrobial cases, enabling complete diagnosis and targeted antibiotic selection (Mei et al., 2023). As a breakthrough technology with unparalleled sensitivity built on high-throughput sequencing, mNGS has garnered increasing attention for infectious disease diagnosis. However, routine use is limited by high costs and variable insurance coverage. Still, the 2018 International Consensus Meeting endorsed mNGS to supplement standard diagnostics for PJI (Shohat et al., 2019). Beyond identifying polymicrobial cases, mNGS has even greater utility in diagnosing culture-negative PJI, which occur more commonly with rare pathogens. In fact, culture positivity was only 53.1% for rare pathogens compared to 73.8% for common bacteria in our cohort. For cases with negative preoperative cultures, we assessed the results of mNGS based on the criteria provided in previous literature (Wang et al., 2024). Thus, mNGS is especially valuable for elucidating rare PJI pathogens. We recommend routine use for suspected PJI with multiple comorbidities, poor health status, or prior invasive procedures to enable timely tailored treatment.

Well-established risk factors for PJI include obesity, diabetes, rheumatoid arthritis, immunosuppression, and cancer (Jämsen et al., 2012; Tande and Patel, 2014; O’Toole et al., 2016). However, differences in predisposing features between rare and common pathogen PJI are less defined. Here, higher CCI and preoperative invasive procedures were more prevalent in the rare pathogen cohort and emerged as independent risks on multivariate regression. Indeed, increasing CCI score raised the likelihood of developing a rare PJI, suggesting certain organisms become opportunistic pathogens in hosts with substantial comorbid conditions like diabetes, renal or liver disease. Meanwhile, breaches to the skin or bloodstream enable invasion of rare organisms residing on the skin or urogenital mucosa into a susceptible joint. Joint aspiration risks direct inoculation if sterile preparation is inadequate. Similarly, preoperative catheterization or venous access may facilitate hematogenous seeding of organisms typically confined to the urethra or skin (Xiang and Lu, 2019). Therefore, we recommend careful consideration of necessity prior to any preoperative invasive procedure.

Laboratory indicators of inflammation like WBC count, CRP, and ESR depend on the host immune response to pathogens. Currently, ESR and CRP continue their role as first-line screening tests (Saleh et al., 2018). The lower CRP levels observed in the rare pathogen PJI group are likely the result of multiple factors. Firstly, some rare pathogens are known for their reduced virulence and immunogenicity. Secondly, the elevated CCI scores in this patient cohort may indicate a compromised capacity to mount an effective immune response to infection. Moreover, it is important to consider the potential impact of various other factors, such as antibiotic use, on these findings.

PJIs caused by distinct organisms have unique antimicrobial susceptibility profiles, necessitating tailored regimens diverging from typical empiric protocols. Moreover, without mNGS, many rare cases would be culture-negative, prompting prolonged broad spectrum or multidrug antibiotics with more adverse events (Cortes-Penfield et al., 2023). Such misguided regimens may also fail to cover the causative organism, jeopardizing infection control. The longer antibiotic durations and higher advent event rates among the rare pathogen cohort stem from several factors. First, comorbid conditions like renal or hepatic dysfunction predispose these patients to drug-related laboratory changes. Second, some initially received inappropriate broad-spectrum antibiotics or combination therapy before the rare organism was detected. Finally, once identified, targeted antibiotics are often continued for prolonged durations to ensure eradication, increasing adverse event risks.

The insidious presentations of rare pathogen PJI coupled with our lack of clinical experience in managing these elusive infections may necessitate prolonged treatment courses and portend suboptimal outcomes compared to typical cases. Previously, one study reported a 25% reinfection rate for rare pathogen PJIs (Anagnostakos et al., 2021). In our current series, the difference of reinfection between the two groups did not reach statistical significance. We attribute our favorable control rate for rare pathogens to the use of mNGS, which expedites the identification of organisms and enables timely, targeted antimicrobial therapy. With protocols to enhance detection and characterize susceptibility, even uncommon organisms may be effectively treated. Nevertheless, vigilance and multidisciplinary engagement remains essential when rare pathogen PJI is suspected. It is suggested that interdisciplinary approaches should be implemented as a standard of patient care to further improve clinical outcomes in the treatment of bone and joint infection (Walter et al., 2022). Orthopedic surgeons should maintain communication with microbiology and infectious disease partners to integrate molecular diagnostics when standard cultures fail, identify ideal targeted regimens, and optimize clinical outcomes for these challenging cases. Ongoing investigation is still needed to clarify best practices.

Despite the findings, several limitations deserve mention. The retrospective nature and single center design, while allowing comparative analysis, introduce limitations in interpretation. The number of rare pathogen PJIs, while sizeable for an isolated experience, remains small from a representative view. The imbalance in sample sizes between the two groups may weaken the strength of the study’s findings. Thus, confirmation with larger, multicenter samples would strengthen conclusions. Additionally, data regarding radiographic characteristics, patient-reported outcomes, satisfaction scores were not available for assessment, representing areas for future investigation. Finally, a few cases had follow-up under two years which, while adequate to capture most recurrences, may underestimate delayed reinfections thereby skewing rates. Long-term monitoring for these cases is ongoing. Nevertheless, this series addresses a highly relevant issue, highlights distinguishing factors to raise clinical suspicion, and suggests timely molecular diagnosis and tailored treatment may negate the notoriously poor prognosis of rare pathogen PJI. Ongoing study to optimize detection, therapy, and prevention will continue to clarify best practices for these elusive PJI causes.

## 5 Conclusion

In summary, PJI caused by rare pathogens represent a distinct subset with some different clinical presentations and risk factors necessitating heightened awareness. Specifically, higher CCI and preoperative invasive procedures should clue clinicians to the possibility of a rare pathogen. In this context, we advocate integrating mNGS to optimized culture methods, leveraging its unbiased detection to facilitate early identification and tailored treatment. Equally important is maintaining open communication across orthopedic surgery, infectious disease, and microbiology teams to integrate molecular findings, select targeted regimens, and optimize outcomes. With protocols tailored to their detection and treatment, even rare pathogen PJI may be effectively controlled, avoiding the dismal prognosis expected for these elusive infections in the past. Nevertheless, further research is still needed to clarify diagnostic and therapeutic best practices.

## Data Availability

The raw data supporting the conclusions of this article will be made available by the authors, without undue reservation.
